# Basalt Fibre Reinforcement of Bent Heterogeneous Glued Laminated Beams

**DOI:** 10.3390/ma14010051

**Published:** 2020-12-24

**Authors:** Agnieszka Wdowiak-Postulak

**Affiliations:** Faculty of Civil Engineering and Architecture, Kielce University of Technology, 25-314 Kielce, Poland; awdowiak@tu.kielce.pl; Tel.: +48-41-34-24-480

**Keywords:** glued laminated beams, timber quality classes, basalt fibre reinforcement, bending strength, theoretical analysis, numerical analysis

## Abstract

The purpose of this paper is to demonstrate the properties of glued laminated beams made in diverse configurations of timber quality classes, reinforced using a new technique that is cheaper and easy to apply. The aim of the experimental investigations was to enhance reinforcement effectiveness and rigidity of glued laminated beams. The tests consisted of four-point bending of large-scale specimens reinforced with basalt fibres (BFRP). The tests were meant to obtain images of failure, the load–displacement relation and load carrying capacity of basalt fibres depending on the reinforcement ratio. The tests, which concerned low and average quality timber beams, were conducted in a few stages. The aim of the study was to popularize and increase the use of low-quality timber harvested from reafforested areas for structural applications. In the study, theoretical and numerical analysis was carried out for reinforced and unreinforced elements in various configurations of wood quality classes. The aim was to compare the results with the findings of experimental tests. Based on the tests, it was found that the load carrying capacity of beams reinforced with basalt fibre was higher by, respectively, 13% and 20% than that of reference beams, while their rigidity improved by, respectively, 9.99% and 17.13%. The experimental tests confirmed that basalt fibres are an effective structural reinforcement of structural timber with reduced mechanical properties.

## 1. Introduction

Timber applications in the construction industry are limited due to the material natural defects, and the necessity to obtain industrially applicable members of proper dimensions [1,2]. Tests have been performed to provide *timber* with *improved* structural *properties.* In the tests, timber was combined with other materials, thus creating composites with improved mechanical properties [1].

Glued laminated beams is used in the construction of long-span lightweight structures. Structural sawn timber layers are joined with adhesives, resulting in increased strength and rigidity [1]. This method allows producing timber elements in which defects are dispersed and the final products have more homogeneous properties. Glued laminated beams allows the production of longer elements that carry higher loads; consequently, it is an efficient and promising structural material.

Reinforcement with FRP (Fibre Reinforced Polymers) materials effectively improves the structural properties of existing timber structural elements. This involves insertion of bars or sheets within timber using epoxy glue or resins [3]. Many tests have demonstrated the structural effectiveness of reinforcing glued timber beams with FRP materials and epoxy adhesives used as bonding. The method allows for obtaining significant bending strength. However, cut-out grooves in long span elements should be of minimal width, thus allowing for achieving the perfect visual effect. An important application of the method is the reinforcement of poor-quality pine timber, as described in Raftery and Rodd [4]. In Gilfillan et al. [5], reinforcement was applied to increase the strength and rigidity of structural sawn timber of poor quality, and was also used in restoration of existing bridges made of glulam or solid timber [6]. Additionally, many studies reported strengthening timber beams with various types of FRP reinforcement (bars, belts, strings, boards) placed at different sites (for example, reinforcement bonded externally to the surface) [7]. The most frequent type of reinforcement is direct installing of bars with a portion of epoxy glue. In order to apply reinforcement of bars, one or several grooves must be cut out alongside the entire length of a beam. Bars should be inserted into the cut-out grooves and bonded with a resin (e.g., epoxy resins). Many researchers [5,6,7,8,9,10,11,12,13,14,15,16,17,18,19,20,21,22,23,24] conducted tests and theoretical analyses of solid and glued timber. Raftery and Kelly [25], for example, reinforced poor- quality glued timber by means of installing basalt FRP bars. The results showed that average rigidity increased by approx. 10% for 1.4% reinforcement applied underneath the ceiling beam.

An important aspect of the present study involves the use of reinforcement of natural fibres (e.g., basalt, flax, bamboo, hemp, etc.) to reduce the environmental effect of the method. The investigations reported in this study aim to restore the original load capacity of the existing structures and to contribute to the design of new ones [26]. Additionally, beams reinforced with natural fibre composites show improved mechanical properties including tensile strength and lightness [27,28]. Other advantages include lower costs, pollutant emission and energy consumption in production and after-use-phase [29,30,31]. It is also important that, compared with synthetic fibres, the costs of production and waste management of natural fibres are much smaller. 

The study [32] reports bending tests on pine beams reinforced with basalt and carbon fibres. The tests showed good mechanical properties of the specimens to which BFRP fibres and bi-directional carbon fabrics were applied. Research was also carried out on the application of basalt NSM (Near Surface Mounted) FRP reinforcement in solid wooden beams [33]. Based on the investigations, it was found that the average increase in load capacity for NSM FRP reinforced beams was 16% compared with unreinforced beams. In study [34], the effect of reinforcing cracked wooden beams (96 × 96 mm^2^ square and 2000 mm long) with carbon and basalt rods (BFRP) was analysed. The reinforcement of wooden beams with CFRP (Carbon Fibre Reinforced Polymers) inserts made it possible to increase the bending strength by 29.4%. Moreover, a diagonal arrangement of the BFRP bars in the wooden beams resulted in an 18.8% increase in bending strength compared with unreinforced beams. The results are comparable to those of Yeboah et al. [35], who obtained increased load-bearing capacity by application of glued BFRP wooden bars, and discussed other advantages of using BFRP rebar in wooden structures. Furthermore, McConnell et al. [36] in their investigations into the reinforcement of wooden beams with BFRP tensile basalt fibres noted an increased load capacity and rigidity of 28% and 8.7%, respectively. In addition, in [37], low-quality glued laminated timber was reinforced with basalt fibre rods, while preliminary tests of wood strengthening with pre-stressed basalt fabric were carried out [38]. It was concluded that further experiments on the possibility of using pre-stressed basalt fabric in glued laminated timber beams should be performed.

In [39], the researchers investigated the durability and mechanical bending properties of basalt fibre used in concrete. Based on accelerated aging tests, it was found that basalt fibre has better resistance than fibreglass. Additionally, reinforcement with basalt fibres was considered a good option for high fire resistance. In [40], tests were carried out on corrosion resistance of basalt and glass fibres in sea water. The tensile and bending strength of the specimens decreased with time when exposed to sea water, but the anticorrosive properties of basalt fibres were similar to those of fibreglass. In [41], researchers explored the effect of elevated temperature on fibre-reinforced beams (FRP) using an electric furnace. It was found that BFRP basalt beams showed better mechanical properties than GFRP glass beams. In [42], experimental and numerical studies concerned the effect of BFRP fabric reinforcement on the shear strength of reinforced concrete beams. It was found that the BFRP fabric provides a significant increase in shear strength. The effect intensified as the number of layers increased. As regards structural wood, few experimental tests have been carried out on the use of basalt fibre reinforcement. However, in [35], researchers determined the peel strength of bonded basalt bars. Based on the research, it was found that the peel load increased with the length of gluing.

Additionally, an important issue is the influence of moisture changes in glued FRP-wood joints [43]. It should be remembered that wood is a hygroscopic material; therefore, it undergoes changes in volume under different relative humidity conditions. However, moisture absorption of FRP materials is much lower compared with wood. Thus, hygrothermal stresses arise at the boundary of the FRP-wood joint located at the site exposed to changing environmental conditions. Consequently, careful selection of adhesive and proper preparation of the surfaces to be glued are important requirements for maintaining FRP adhesion to wood [44]. Raftery et al. [44] presents experimental studies on the determination of the adhesion and the influence of moisture content in five adhesives available on the market when combining FRP materials with wood. Two phenol resorcinol formaldehydes (PRFs), a melamine urea formaldehyde (MUF), a polyurethane (PU) and an emulsion polymer isocyanate (EPI) were the adhesives selected [44]. Based on the research, it was found that the adhesion of the connection depended not only on the type of adhesive, but also on the type of FRP reinforcement. Studies of wood and glued bars combinations began with single bars subjected to axial tension [35,45,46]. Preliminary tests provided the description of several different failure modes: bar failure in tension depending on the bar material, adhesion failure of the adhesive related to its strength, and local shear failure of the wood around the bond. The study took into account geometric parameters and strength of the wood, and the failure of the wooden element depending on the type of wood and distance from the edge. In addition to the issues mentioned above, Harvey and Ansell [47] investigated the effect of rod surface preparation, adhesive layer thickness, GFRP rod anchorage length, type of adhesive, type of wood and wood moisture on the load bearing capacity of timber joints. Their results indicated that adhesive in joints should have a thickness of at least 2 mm, but further increase in thickness does not lead to an increase in strength. The study [48] describes tests on specimens made of BFRP bars with a diameter of 12 mm and a thickness of 2 mm, glued with epoxy adhesive over a length of 80 mm to 280 mm, combined with low-quality wood. On the basis of the tests, a significant increase in tensile strength was found with an increase of the gluing length.

This paper advocates the use of poor-quality timber collected at re-afforested areas for structural applications. It also shows the feasibility of BFRP use to enhance load carrying capacity. The paper also reports tests on new glulam beams reinforced with basalt fibre composite materials. The analysis described below is based on a series of tests performed on basalt–reinforced beams, the final dimensions of which were 82 × 162 × 3650 mm^3^. Basalt fibres were used due to low price, which would contribute to a wider use in construction. The aim of the work was to analyze the static work of bent glued beams made of low-quality construction timber for the obtained reinforcement result. The study considered the influence of the systems and degrees of reinforcement on the load-bearing capacity of structural elements, and the nature of the reinforcement in the place of wood defects was also determined. An important advantage was the determination of the impact of the heterogeneity of the structure on the effect of their reinforcement in beam elements, as well as examining the possibility of using lumber of a lower quality class for beams reinforced with BFRP fibres. Additionally, the article contains theoretical and numerical models enabling the analysis of the bearing capacity and stress of beams reinforced with BFRP fibres. Well, there is a growing shortage of high-quality assortments and the need for the necessary economical management of scarce raw material. Therefore, after the conducted analyses, due to the favorable properties of BFRP fibres and their impact on the increase in load capacity, it was found that it was necessary to conduct further research on strengthening of wooden beams using assortments of the lowest classes. So far, no such research attempt has been described in the literature. This program requires an analysis of the structural and geometric features of the construction sawn timber used, divided into wood quality classes. In the literature, the number of tests carried out on BFRP basalt reinforcement in wood engineering is still scarce. Basalt fibre BFRP rods were used as internal reinforcement to strengthen low-quality glued laminated timber [37]. Preliminary studies were also carried out to evaluate the effectiveness of wood strengthening with pre-stressed basalt fabric [38]. It was found that more research is needed in this area. The study [25] described the use of BFRP basalt fibre reinforced bars to strengthen and repair low-quality glued laminated timber. It was shown that, when using a low rate of reinforcement, namely 1.4%, basalt BFRP bars can show an increase in the rigidity by more than 10%, and in the maximum bending moment by more than 23%. The dimensions of glued beams in the test were 96 × 190 mm^2^, and the distance between the supports was 3420 mm.

## 2. Materials and Methods 

### 2.1. Specifications of Materials Used in Glulam Beams

Structural sawn timber was made of the tree species called *Pinus sylvestris* L. The wood was harvested from the Lesser Poland Forest Region at the beginning, and the end of the vegetation period. Sawn timber was then sorted in terms of size and origin, and the moisture was measured with a hygrometer. Next, the elements were labelled, stacked and packaged and transported to a dry kiln in order to obtain average moisture content of 12% [14]. The moisture content was then measured once again for each piece of sawn timber following the standards PN-EN 13183-2:2004 [49] and PN-EN 13183-3:2007 [50]. In accordance with PN-D-94021:2013-10 [51], the test material was inspected visually. The following were given attention: knots, twisted fibres, cracks, resin galls, inbarks, galls, necrosis, blue stain, wood decay, insect holes, reaction wood, average tree ring width, density, wanes, lengthwise curvatures of sides, lengthwise curvatures of planes, width-oriented transverse curvatures or width-oriented warping. Coniferous structural sawn timber, intended for beams, was divided into the following Polish quality grades: KS (average quality), and KG (poor quality). The beams, divided into two groups, originated from two different batches of poor and good quality, the wood density was 420.09 kg/m^3^.

The specifications of GL20c class of glued laminated timber were adopted for beam fabrication. All characteristic mechanical properties, shown in Table 1, are in compliance with PN-EN 14080:2013-07 [52]. The average modulus of elasticity along the fibres and the bending strength, determined on the basis of laboratory tests in accordance with PN-EN 408 + A1:2012 [53], are also given in Table 1.

For test purposes, glulam beams made of components of different timber quality class configuration (KS and KG) were reinforced with basalt fibres (BFRP). Each glulam beam consisted of four lamellas, approx. 40 mm thick each, with total final height of approx. 162 mm. Each lamella was cut out from sawn timber pieces 4000 mm in length. Lamellas were bonded using D4 polyvinyl acetate adhesive (density of 1.10 g/cm^3^, viscosity of 13,000 mPa × s). The characteristics of the materials are collated in Table 2.

In the tests, FRP reinforcement was applied using epoxy adhesive and BFRP basalt fibres with a diameter of 10 mm. The epoxy resin-based adhesive layer was obtained as a result of mixing LG 815 epoxy resin (density of 1.13 ÷ 1.17 g/cm^3^, viscosity of 1100 ÷ 1300 mPa × s) with HG 353 hardener (density of 0.98 g/cm^3^, viscosity of 100 ÷ 150 mPa × s). After mixing the resin with the hardener, the epoxy glue had a bending strength of 0.11 ÷ 0.12 GPa and a modulus of elasticity of 2.7 ÷ 3.3 GPa. The values of moduli of elasticity and final deformation of BFRP basalt fibres were E = 78 GPa and Ɛu = 39%, respectively. The mechanical properties of basalt fibres and epoxy glue are summarised in Table 3.

The effect of moisture on the adhesion of BFRP fibres to glued laminated beams was determined on the basis of the high moisture content of the reinforced glued beams for a period of 2 months. Based on these tests it was observed that the BFRP fibres did not become debonded from the adhesive, and the adhesive did not lose adhesion to the wood.

### 2.2. Preparation of BFRP Reinforcement

**Figure 1:**
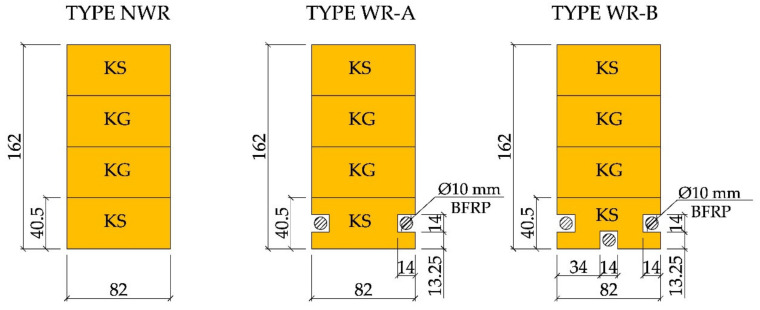
Cross-sections of the tested WR beams [dimensions in mm, KS—average quality grade, KG—poor quality grade].

For BFRP reinforcement, two or three square 14 × 14 mm^2^ grooves were cut into the tensile area of each sample (see Figure 1). Reinforcement in all grooves had an approx. 2 mm cover and was installed using the epoxy glue described above. Prior to glue application, the BFRP materials were purified with “Aceton” solvent. The BFRP basalt fibres were then anchored and pre-stressed (5 mm sheet metal, nuts) and the epoxy glue (LG 815 + HG 353) was applied, filling the square grooves alongside the entire beam length.

### 2.3. Tests

**Figure 2:**
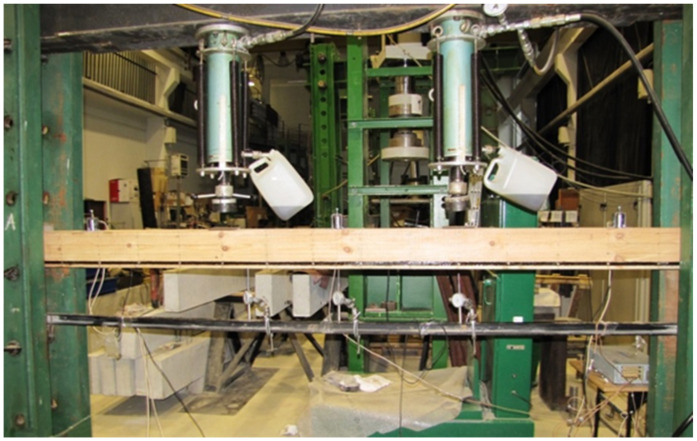
The workstation for WR-A3 beam testing (photo by: Wdowiak-Postulak).

**Figure 3:**
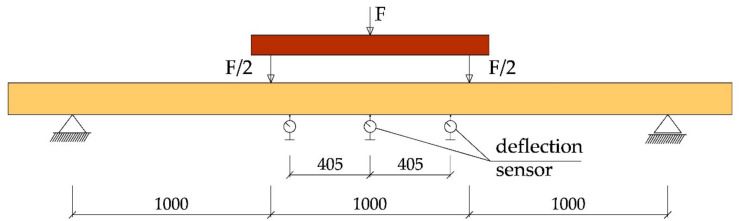
Test workstation diagram (dimensions in mm).

The tests were performed at the Department of Strength of Materials and Analysis of Building Structures of the Kielce University of Technology. The test workstation is shown in Figure 2. Two VEB Werkstoffprufmaschinen Leipzig actuators with piston area of 50 cm^2^ and maximum applied pressure of 10 MPa were used for the tests. Three types of beams (NWR, WR-A, WR-B) were made which differed in the reinforcement ratio. Three beams of each type were analysed. The beams were tested under four-point monotonic loading until failure. All beams had clear spans of 3000 mm (see Figure 3). 

**Figure 4:**
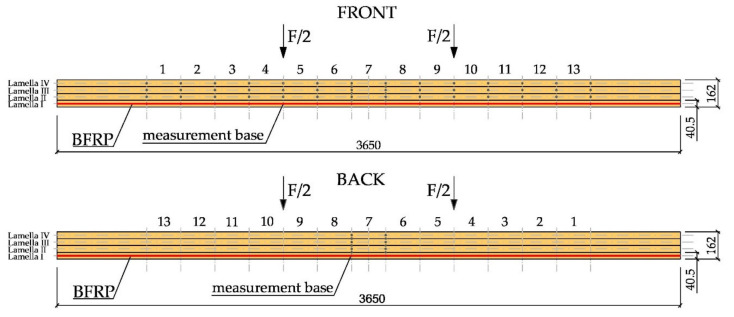
Locations of measurement bases on the front and the back surface of WR-A reinforced beams (dimensions in mm).

The tests were performed in compliance with PN-EN 408 + A1:2012 [53] and the following values were recorded: loading force, beam displacement at the midspan and along 5 h (h—beam section height), strain in timber, strain in fibre composite materials, and the failure load. The failure mode in the tested beams was also specified. Mechanical sensors (Figure 3) were used to measure the maximum absolute deflection, while the “Demec” type mechanical extensometer with fixed measurement base (Figure 4) was used for strains in timber and basalt fibres. Figure 3 shows the arrangement of three mechanical sensors along the length of 5 h, where h represents the beam section height.

Beam testing included four-point bending, two-point loading forces F/2 applied at the distance of 1 m and two supports. For each configuration (beam types: NWR, WR-A, WR-B), tests until failure were performed. The tests comprised three series. The workstation diagram is shown in Figure 3. 

Additionally, strain measurement bases were located 203.2 mm from each other along the entire beam length, in the centre of lamellas, in the tension (lamella I and lamella II) and compression zone (lamella III and lamella IV), on the side surface. On each lamella (lamellas I, II, III and IV), 13 measurement bases were found altogether, which gave a total of 52 measurement bases on one side of the beam. In Figure 3, the details of the arrangement of the measurement base systems can be seen. Figure 4 shows the front of a 3650 mm long beam, on which 13 measurement bases (13 × 203.2 mm) were installed over the length of lamellas I, II, III and IV (the height of a single lamella is 40.5 mm) along with 13 measurement bases on basalt fibre BFRP. Additionally, Figure 4 shows the back of the 3650 mm long beam, on which the measurement bases at the midspan of lamellas I, II, III and IV were marked, accompanied by the measurement bases on the basalt BFRP fibre sites.

### 2.4. Analytical Procedure

American standards: US standard ICBO/Uniform Building Code 5100 [54] and US standard ICBO/Uniform Building Code 6046 [55] were used to determine the load capacity and rigidity of the sampled glued laminated timber reinforced with FRP fibres in the area of the fibres in tension. 

When determining the position of the axis of inertia of the section in bending, the following assumptions were taken into account:

- the moduli of wood elasticity along the fibres in compression and in tension were distinguished,
- the modulus of elasticity of wood under tension was reduced to the modulus of elasticity of wood compressed along the fibres with the coefficient:
  (1) $$n=\frac{E_{t,0,mean}}{E_{c,0,mean}}$$
- the modulus of elasticity of the BFRP fibres in tension was reduced to the modulus of elasticity of the wood compressed along the fibres using the coefficient:
  (2) $$n^{\prime }=\frac{E_{BFRP}}{E_{c,0,mean}}$$
  where:
  $E_{BFRP}$—modulus of elasticity of BFRP fibres in tension,
  $E_{t,0,mean}$—the average modulus of wood elasticity when tensioned along the fibres,
  $E_{c,0,mean}$—the average modulus of wood elasticity in compression along the fibres.

**Figure 5:**
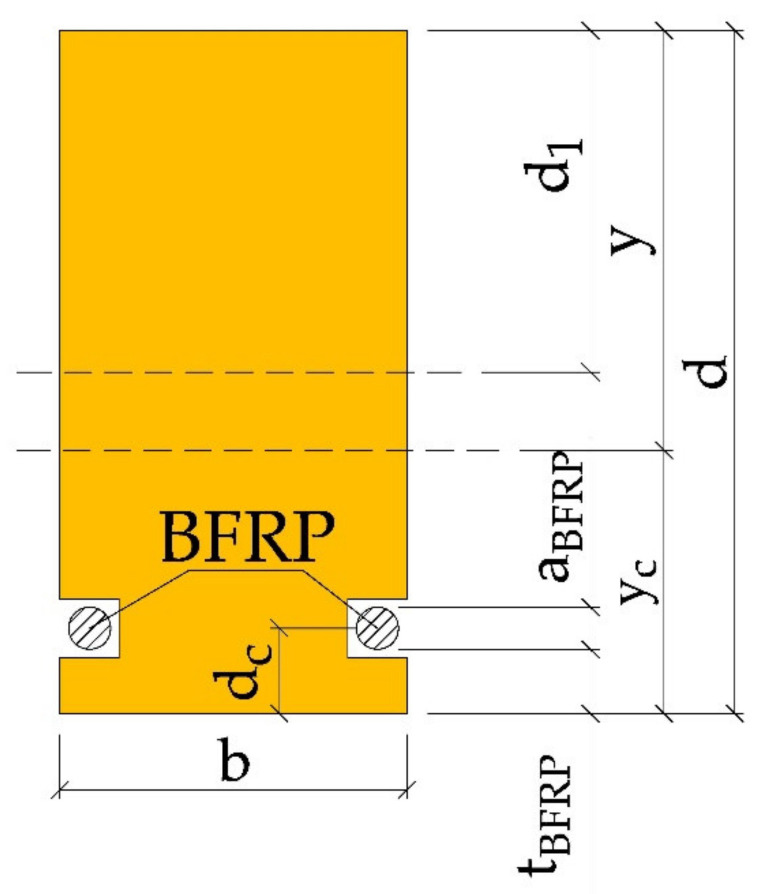
BFRP-reinforced beam cross-section geometry according to US standard ICBO/Uniform Building Code 5100 [54] and US standard ICBO / Uniform Building Code 6046 [55], (b—section width, $d_{c}$—BFRP reinforcement distance to the lower edge of the beam, $d_{1}$—distance of the neutral axis to the beam top before correction for reinforcement, $a_{BFRP}$—BFRP fibre diameter, y—distance of the neutral axis after reinforcement to the upper edge of the beam, $y_{c}$—distance of the neutral axis to the lower edge of the beam, $t_{BFRP}$—thickness of the “cover” of the BFRP fibre, d—the height of the glued beam).

**Figure 6:**
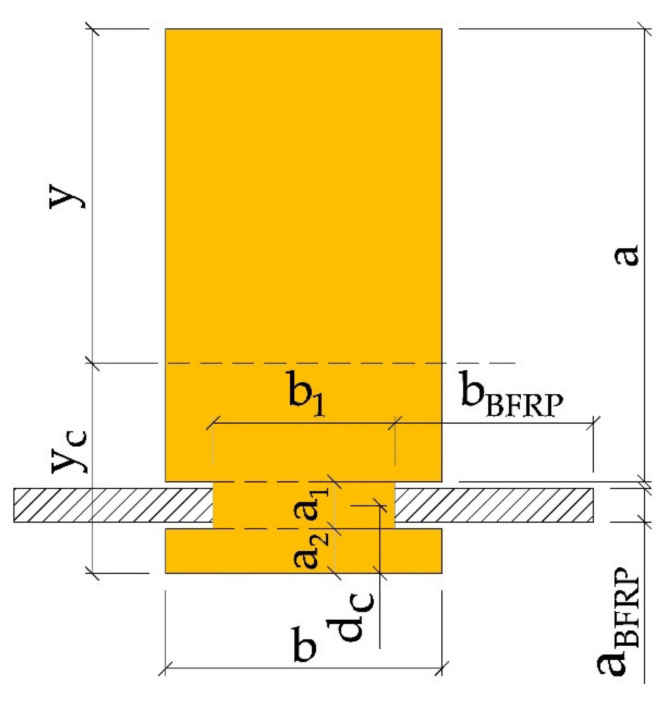
Calculation diagram of a beam reinforced with BFRP fibres (according to the Steiner theorem, the figure has been divided into smaller sections, as shown in the drawing, including a—height of the first section, b—width of the first section, $a_{1}$—height of the second section, $b_{1}$—width of the second section, $a_{2}$—height of the third section, $b_{1}$—width of the third section, $a_{BFRP}$—the diameter of the BFRP fibre, $b_{BFRP}$—the width of the BFRP fibre, y—distance of the neutral axis after reinforcement to the upper edge of the beam, $y_{c}$ —distance of the neutral axis to the lower edge of the beam, $d_{c}$—BFRP reinforcement distance to the lower edge of the beam).

**Figure 7:**
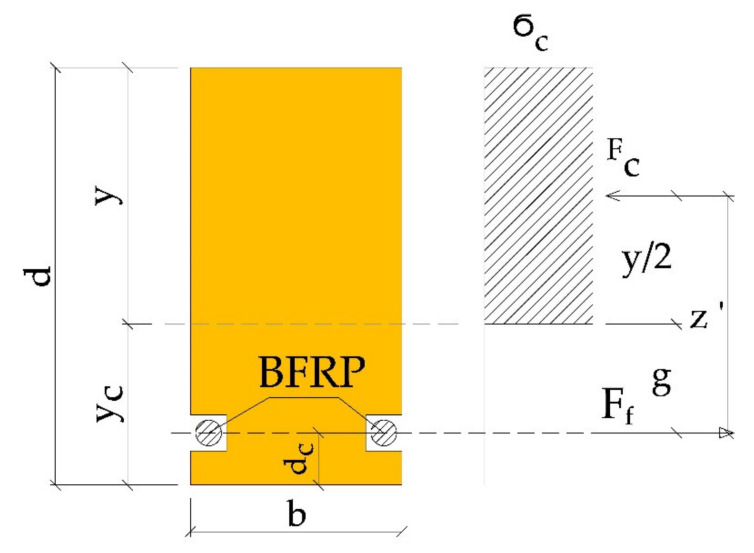
Calculation according to US standard ICBO/Uniform Building Code 5100 [54] and US standard ICBO/Uniform Building Code 6046 [55], (where g—distance of the centre of gravity of the tension reinforcement from the neutral axis, z′—internal forces arm, $F_{c}$—compressive force, $F_{f}$—force transmitted by BFRP, $\sigma_{c}$—compressive stress, d—the height of the glued beam, y—distance of the neutral axis after reinforcement to the upper edge of the beam, $y_{c}$—distance of the neutral axis to the lower edge of the beam, $d_{c}$—BFRP reinforcement distance to the lower edge of the beam, b—width of the first section).

The height of the compression zone y with sole reinforcement of the tension zone (Figure 5, Figure 6 and Figure 7) is:

(3) $$y=d_{1}+M_{t}-N_{t}$$

where:

(4) $$d_{1}=d\frac{\sqrt{n}}{1+\sqrt{n}}$$

(5) $$N_{t}=nd_{c}\left(n^{\prime }-1\right)\frac{a_{BFRP}}{d_{1}}$$

(6) $$M_{t}=n{\left(n^{\prime }-1\right)}^{0.63}a_{BFRP}$$

where:

$M_{t}$—adjustment value for reinforcement in tension zone,
$N_{t}$—adjustment value for bumper layer in tension zone,
d—the height of the glued beam,
$d_{1}$—distance of the neutral axis to the beam top before correction for reinforcement,
$a_{BFRP}$—BFRP fibre diameter,
$t_{BFRP}$—thickness of the “cover” of the BFRP fibre,
$d_{c}$—BFRP reinforcement distance to the lower edge of the beam,
y—distance of the neutral axis after reinforcement to the upper edge of the beam,
$y_{c}$—distance of the neutral axis to the lower edge of the beam.

The position of the neutral axis with respect to the lower edge of the glued beam can be determined from the formula:

(7) $$y_{c}=\frac{\left(ab\right)\left(\frac{a}{2}+a_{1}+a_{2}\right)+\left(a_{1}b_{1}\right)\left(\frac{a_{1}}{2}+a_{2}\right)+\left(a_{2}b\right)\frac{a_{2}}{2}+2\left(\left(a_{BFRP}b_{BFRP}\right)d_{c}\right)}{\left(ab\right)+\left(a_{1}b_{1}\right)+\left(a_{2}b\right)+2\left(a_{BFRP}b_{BFRP}\right)}$$

The moment of inertia $I_{Z}$ of the inserted section can be specified using the Steiner theorem:

(8) $$\begin{array}{l} I_{z}=\left(\frac{ba^{3}}{12}+ab{\left(\left(\frac{a}{2}+a_{1}+a_{2}\right)-y_{c}\right)}^{2}\right)+\left(\frac{b_{1}a_{1}^{3}}{12}+a_{1}b_{1}{\left(y_{c}-\left(\frac{a_{1}}{2}+a_{2}\right)\right)}^{2}\right)\\ +\left(\frac{ba_{2}^{3}}{12}+a_{2}b{\left(y_{c}-\frac{a_{2}}{2}\right)}^{2}\right)+2\left(\frac{b_{BFRP}a_{BFRP}^{3}}{12}+b_{BFRP}a_{BFRP}{\left(y_{c}-d_{c}\right)}^{2}\right) \end{array}$$

where:

(9) $$h_{t}=d-y-t_{BFRP}-a_{BFRP}$$

(10) $$y_{c}=d-y$$

In the American standards, the load capacity of the reinforced cross-section is determined assuming full plasticization of the compressed zone of the wood. Additionally, the bearing capacity is determined by the strength utilisation of the compression zone. In determining the load capacity, the share of wood in the area of tensioned fibres is ignored. Tensile stresses are assumed to be transmitted by the BFRP fibres.

Taking into account the above assumptions, the allowable moment resistance of the cross-section is equal to (see Figure 7):

(11) $$M_{r}=F_{c}z^{\prime }$$

where:

(12) $$F_{c}=\sigma_{c}yb$$

(13) $$z^{\prime }=\frac{y}{2}+g=\frac{y}{2}+\left(y_{c}-d_{c}\right)=d-\frac{y}{2}-d_{c}$$

Tensile stresses in BFRP fibres were determined according to the formula:

(14) $$\sigma_{BFRP}=\frac{M_{r}\left(g+\frac{a_{BFRP}}{2}\right)n^{\prime }}{I_{z}}$$

### 2.5. Numerical Procedure

Wood is both an anisotropic material (properties depend on the direction, in one direction it has different properties than in the other) and a heterogeneous material.

The numerical analysis of glulam timber beams composed of various wood quality classes was carried out in the ANSYS 16.0 environment, using the Static Structural module. Both unreinforced and reinforced beams were modelled for numerical analysis. The geometry and load systems of the model were adopted in accordance with the results obtained for experimentally tested beams. The dimensions of beams were 82 × 162 × 3650 mm^3^, and each beam consisted of four 40.5 mm thick lamellas. Tested glulam beams were made from the tree species called *Pinus sylvestris* L. The basalt fibres BFRP and epoxy glue (LG 815 + HG 353) were used to strengthen the beams. 

The geometrical models of the beams were made in CATIA V5 as a combination consisting of the following elements: blocks constituting the supports and points of application of loading forces, lamellas, BFRP fibres and an adhesive filling the space between the lamella and BFRP fibres. 

**Figure 8:**
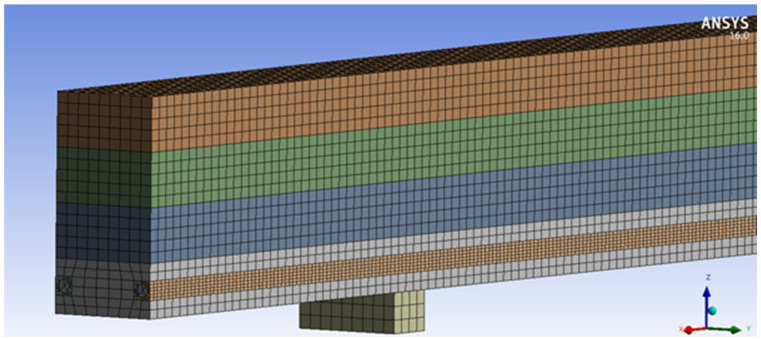
Finite element mesh of WR-A beam.

Timber, BFRP and epoxy were modelled as finite elements. The sizes of the elements were adopted on the basis of the mesh discretisation test. The finite element mesh used for the analysis is shown in Figure 8. The finite element mesh consisted of hexagonal and tetragonal elements. The lamellas and supports were modelled as hexagonal elements with a size of 10 mm. Due to the small dimensions of the BFRP fibres and the epoxy adhesive layer in relation to the rest of the geometry, they were defined as tetragonal elements with a size of 5 mm. In the analysis, the glued joint between successive lamellae was considered to be a bonded joint (see Figure 8).

The lamellas of the glulam beams were modelled as separate parts so that each of their material properties could be taken into account. Thus, 4 lamellas of different quality classes were modelled (see Figure 1), lamellas I and IV as the KS wood quality class, and lamellas II and III as the KG wood quality class. It was assumed that there was a sufficient bond between the lamellas and the adhesive layer was not modelled due to its very small thickness. In lamellas I, square holes were modelled and filled with BFRP fibres and epoxy glue (see Figure 8). The interphase regions between *wood* and *epoxy, and also* epoxy glue and BFRP were considered to have sufficient bonding, as experimental studies confirmed the satisfactory quality of the joints.

In the investigations, a three-dimensional FE model was defined. It was employed to determine the behaviour of unreinforced and reinforced beams made in different configurations of wood quality classes. The reinforcement employed was BFRP basalt fibres. The dimensions of the elements, as well as the loading diagrams, were analogous to those found in the laboratory tests. The numerical tests included checks and comparison of normal stresses, deflections of unreinforced beam elements and reinforced ones in laboratory and numerical analysis.

In order to obtain accurate results of numerical analysis, all materials involved should be modelled correctly. As wood is anisotropic, material parameters should be determined for different directions in the material. Nine independent constants (three moduli of elasticity, three shear moduli and three Poisson’s ratios) were used to describe the mechanical properties of the lamellas (Table 4). 

Material parameters of wood and basalt fibres BFRP in numerical tests were adopted based on the author’s research and literature data (Table 4 and Table 5), [52,56,57]. In the experimental tests, the modulus of elasticity along the fibres was determined. Poisson’s ratios for wood were adopted in accordance with [56], while other values were determined on the basis of PN-EN 14080:2013-07 [52], PN-EN 338:2016-06 [57]. Specifications for BFRP basalt fibres and epoxy adhesive were determined on the basis of the manufacturer’s data. The numerical analysis assumed that the material properties are independent of the load factors. As a result, relative humidity, temperature and other environmental factors were not taken into account in this numerical model. 

Wood and reinforcement were defined as orthotropic materials, whereas the adhesive was considered to be isotropic material.

The following parameters were adopted for the analysis:

- wood quality class KS: T13 (C22),
- KG wood quality class: T8 (C14).

## 3. Results

The analysis of the results obtained in tests of glulam beams is described below. The beams were made of *Pinus sylvestris* L., harvested from the Lesser Poland Forest Region, and reinforced with BFRP basalt fibre. The subchapters deal with the following: force-bending relation, strain analysis and normal stress analysis, images of failure, and comparison of theoretical and numerical analysis with laboratory results. 

### 3.1. Force-Bending Relation

**Figure 9:**
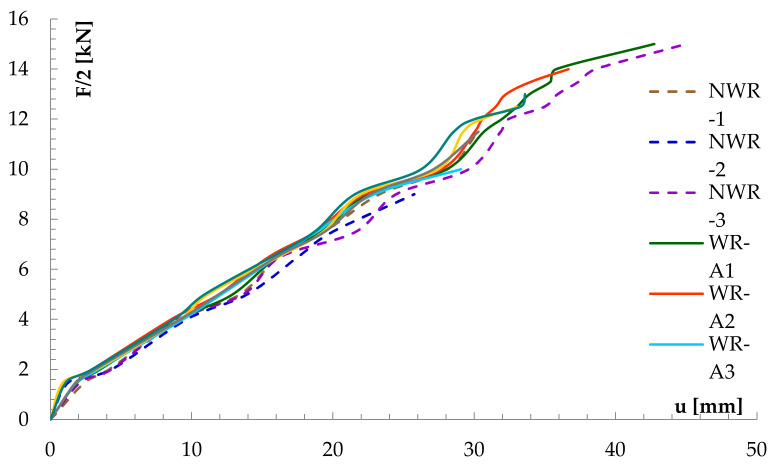
The diagram of the F/2—u relation in the WR beams tested as read from sensor 2 (beam midspan). Note that the measurements stop when sensors were removed before beam failure.

As described above, the NWR beam was a reference for the reinforced WR-A and WR-B beam types. Figure 9 shows the deflection and loading curves for the beams of concern obtained from sensor 2.

It can be seen that pre-stressed BFRP basalt fibre effectively reduced increase in deflection values at the midspan. For all glulam elements in reinforced and unreinforced beams, the average deflection value for 10 kN load was approx. 12.43 mm. Figure 9 shows the efficiency of basalt fibres reinforcement in all the tested glulam beams for different loads applied. Rigidity of WR-B beams (reinforcement ratio of 1.76%) was visibly greater than that of WR-A beams (reinforcement ratio of 1.18%). At 1.76% BFRP reinforcement in laminated beams, the increase in splint was about 17.13%, while, at 1.18% BFRP reinforcement, this increase was around 9.99%. It should be noted that, prior to beam failure, mechanical sensors were removed to protect them from damage.

### 3.2. Analysis of Normal Stresses

**Figure 10:**
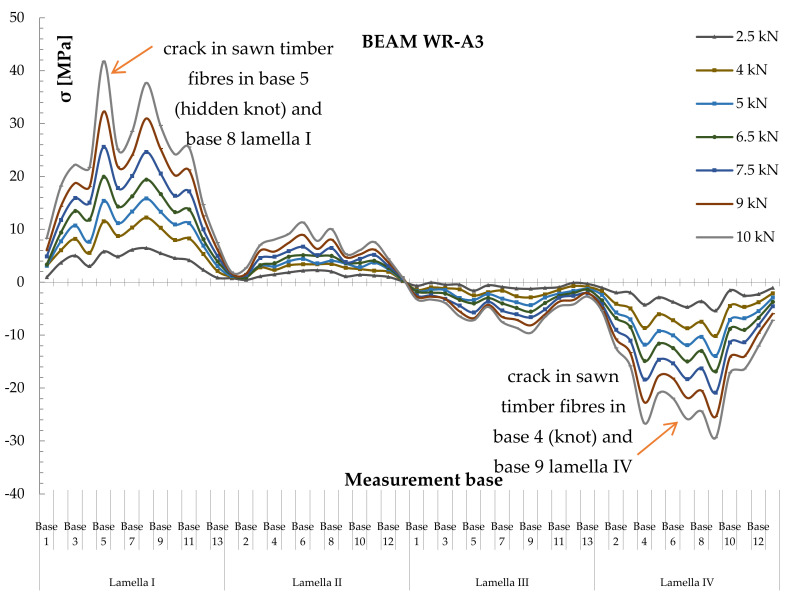
Distribution of normal stresses in timber σ [MPa] along the entire length of the rein Figure 3 beam.

**Figure 11:**
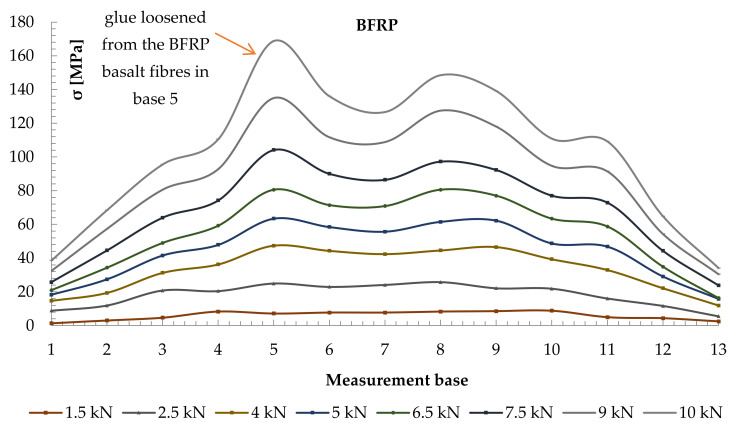
Distribution of normal stresses in basalt fibres BFRP Ϭ [MPa] along the entire length of the reinforced WR-A3 beam.

Strain values were recorded by the sensors located in the measurement bases on the section side surfaces in the beams located 203.2 mm from each other (see Figure 4). Normal stresses were then analysed. To determine normal stresses in timber, the modulus of elasticity of the value of 10.3 GPa, calculated in experimental tests, was applied. Diagrams of normal stress distribution, together with timber defects, are shown in Figure 10 and Figure 11. Two typical patterns of normal stress distribution in WR-A3 beams can be seen in the figures. Figure 10 shows the distribution of normal stresses σ [MPa] in the timber over the entire length of the WR-A3 reinforced beam for different loads F/2 [kN]. The distribution of normal stresses in four lamellas of the glued beam is shown against the spacing of measurement bases (Figure 4). In Figure 10, lamellas I and II experience tensile stresses, while lamellas III and IV are affected by compressive stresses along the entire length of the glued beam. The graphs of stress distribution made it possible to show the lowering of the beam neutral axis after reinforcement WR-A3. Figure 11 shows the values of normal stresses along the entire length of basalt fibres (13 measurement bases) in the tension zone. For the determination of normal stresses in BFRP fibres, the elastic modulus of BFRP was applied. The modulus value of 56.3 GPa was calculated in experimental tests.

Normal stresses diagrams covered various loading phases from the elastic phase until the instant of beam failure. Based on the diagrams of normal stresses distribution, it can be seen that the BFRP beam reinforcement is advantageous for a lowering of the neutral axis. This leads to increased stress in the compression zone that could result in beam plasticization. It was found in the tests that stress values in the timber section were smaller for beams reinforced with basalt fibres compared with unreinforced beams.

It should be noted that asymmetric distributions of normal stresses, as shown in Figure 10 and Figure 11, were caused mostly by timber defects, usually cracks in knots, wood fibres, etc. This was particularly visible in average- and poor-grade materials. In contrast, basalt fibres took over the tensile forces; consequently, existing stress increased. The observed effects of the increase in normal stresses in wood and in basalt fibres in individual measurement bases are shown in Figure 10 and Figure 11.

The results of the experimental tests are summarized in Table 6. The observed variation values, even if the number of samples is limited, give a clear indication of the uncertainty of the measured data.

### 3.3. Modes of Failure

**Figure 12:**
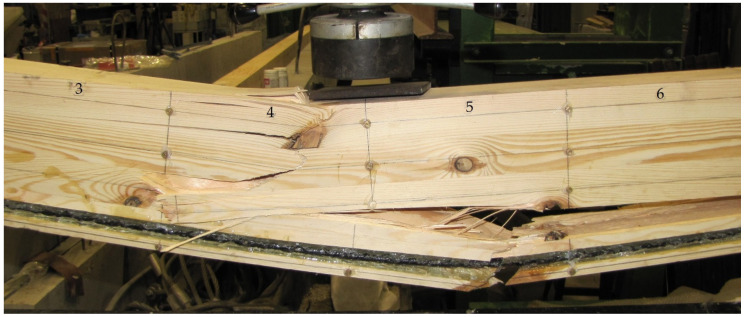
Failure of the tension and compression zone of the glulam beam reinforced with basalt fibres—the WR-A3 beam (3, 4, 5, 6—measurement bases, photo by: Wdowiak-Postulak).

Images of failure were different for individual beams. Failure of beams resulted mostly from heterogeneous timber structure, such as fibre defects (knots) or faults. In Figure 12, the image of failure in the WR-A3 beam can be seen. Failure in the compression area (base 4, crushing, hidden knot) and the tension area (bases 4–9, cracked timber fibres) occurred for the 34 kN load. Locations of individual measurement bases are shown in Figure 4. Failure of the glulam timber beam began with the crushing of timber in the compression zone, which resulted in the lowering of the neutral axis, followed by plasticization. This provided an additional safety margin during failure. 

### 3.4. Comparison of Theoretical and Numerical Analysis with Laboratory Tests

**Figure 13:**
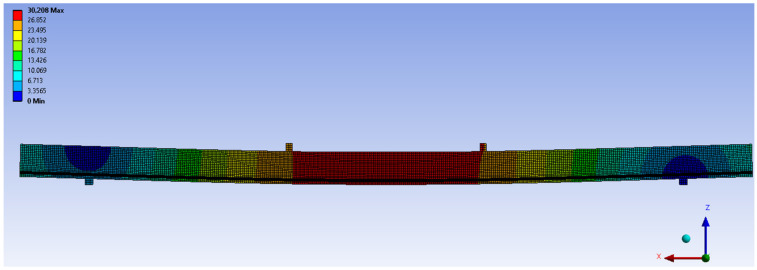
Image of displacements u [mm] from Ansys software for the force F/2 = 10 kN in WR-A beam series.

Numerical and theoretical tests included checks and comparison of bending and normal stresses in wood and BFRP fibres in beam elements with laboratory results. An exemplary image of displacements, obtained from the Ansys program for the WR-A beam series, is shown in Figure 13. In the numerical studies, a three-dimensional finite element model was defined. It was used to determine the behaviour of unreinforced and reinforced beams, made in various configurations of wood quality classes, reinforced with BFRP basalt fibres.

**Figure 14:**
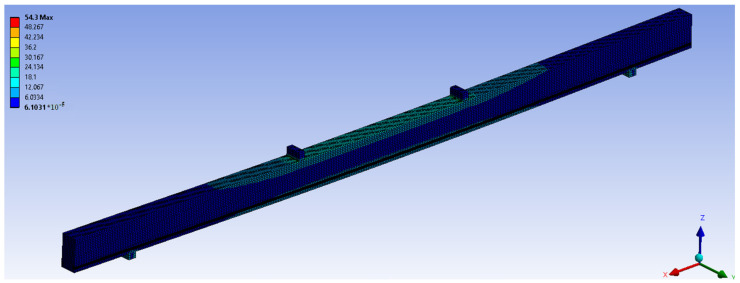
Stresses in wood and basalt fibres BFRP from Ansys software for the force F/2 = 5 kN in WR-A beam series.

The following beam types were analysed numerically: NWR, WR-A and WR-B. The dimensions of the elements, and the loading diagrams were analogous to those in the laboratory tests. Numerical tests included checks and comparison of normal stresses and displacements of unreinforced beam elements with those of reinforced elements. Figure 14 shows the view of the numerical model of stresses in wood and basalt fibres BFRP from Ansys software for the force F/2 = 5 kN in WR-A beam series.

In the paper, FEM was employed to simulate a composite beam reinforced with BFRP fibres. The correlation between the numerical and experimental results was discussed. The experimental, theoretical and numerical results were compared to verify the accuracy of the analytical values and the FE models. Exemplary results are collated in Table 7.

As can be seen in Table 7, the analytical values were close to the laboratory ones. For the exemplary glued beams, the difference in the normal stresses in basalt fibres BFRP (σ_BFRP_) was mainly caused by the variation in the properties of the structural timber. It can be concluded that the structural and geometric heterogeneity were the main reasons for this significant difference. All theoretical results exceeded the experimental and numerical results.

As seen in Table 7, the numerical analysis of the values of displacements, normal stresses in timber and basalt fibres BFRP (σ_BFRP_) showed good correlation with the laboratory analysis. For all glued reinforced and unreinforced elements, the series of NWR and WR beams (medium and lower quality class) showed high compliance with the experimentally determined displacements distribution. The differences ranged from 7 to 23% in relation to the laboratory analysis. The numerical analysis of the normal stress values for timber and BFRP basalt fibres also showed a similar correlation to the laboratory analysis (see Table 7). Differences in the values of compressive stresses σ_c_ for glued timber in relation to laboratory tests ranged from 2 ÷ 21%, while in the values of tensile stresses σ_t_ 8 ÷ 34%, stresses in BFRP fibres were 8 ÷ 12%. The stress values in the BFRP fibres were almost equivalent. It should be remembered that the differences between the results obtained from the numerical analysis and the laboratory tests may be the result of the simplifications used in the analysis. Wood is a complex organic material with an anisotropy of mechanical properties. Due to the limitations in computer modelling and numerical analysis, it is impossible to include all the structural complexities of wood (e.g., irregularities in construction, wood defects) in the study. The results obtained from the numerical analysis show a high congruence with the experimental results when the materials were defined through the actual values obtained from experimental tests corresponding to each wood quality class. Therefore, it is advisable that researchers should carry out materials tests by themselves and determine mean values for each quality class of structural timber.

## 4. Conclusions

The paper reports bending tests for glulam beams made of *Pinus sylvestris* L., harvested at the Lesser Poland Forest Region at the beginning and the end of the vegetation period, reinforced with basalt BFRP fibres. The study also includes analytical and numerical results to compare them with laboratory tests. The most important conclusions to be drawn from this research paper are as follows:

- It was noted that the failure load significantly increased with an increase in the reinforcement ratio, while maximum bending was slightly reduced. Based on the test result analysis, it was concluded that the load capacity of the WR-A and WR-B was increased by, respectively, 13% and 20%, while their rigidity grew by 9.99% and 17.13%, when compared with reference beams. The tests also demonstrated that application of BFRP reduced variation in the bending strength.
- In the tests, it was found that basalt fibres perfectly compensated for the heterogeneous timber structure, being an ideal application to timber of poor and average quality. BFRP fibres inhibit or limit the propagation of cracks, neutralizing local defects in the wood. An important conclusion is that glulam beams made mostly of sawn timber of poor and average quality and reinforced with basalt fibres have better strength and rigidity parameters, and their failure modes show an increase in the elastic phase.
- It should be remembered that BFRP reinforcement in glulam beams can be applied both on-site and in the beam manufacturing process. Timber gluing is an effective method that allows for obtaining more uniform stress distribution between reinforcement and timber. The 2 mm thick epoxy layer, located directly between reinforcement and timber, maintained good quality during the entire test period. No premature delamination between the FRP composite material and timber was detected before complete timber failure.
- Theoretical analysis is important for the designer to select the appropriate type of reinforcement when designing new reinforcement techniques. For the adopted computational model of BFRP fibre composites reinforced beams in bending, the values of normal stresses of BFRP fibres were slightly higher than in laboratory or numerical tests.
- Displacements, normal stresses of wood and BFRP basalt fibres were determined with reasonable accuracy. A three-dimensional FE model was consistent with the experimental studies. The findings can be useful for further analysis of the reinforcement and repair system employing BFRP fibres. The numerical analysis can be applied to reinforced elements when designing various reinforcement schemes, with particular emphasis put on the configuration of wood quality classes. However, it is necessary to carry out material tests of the analysed elements on individual basis, and to determine the mean values for each quality class of structural timber. The determination of Poisson’s ratios for particular strength classes would allow for conducting analysis of structures with advanced computational systems at the stage of structure design.

## Figures and Tables

**Table 1:** Mechanical properties of GL20c glued beams.

Properties	According to PN-EN 14080:2013-07	According to PN-EN 408 + A1:2012
Bending strength (MPa)	20	23.31
Tensile strength (MPa)		
along the fibres	15	-
across the fibres	0.5	-
Compressive strength (MPa)		
along the fibres	18.5	-
across the fibres	2.5	-
Modulus of elasticity (MPa)		
along the fibres	10,400	10,300
Shear-modulus (MPa)	650	-
Density (kg/m^3^)	355	420.09

**Table 2:** Characteristics of beam types of average and poor quality grades, reinforced with BFRP.

Beam Type	Description
NWR	non-reinforced beams made of glulam timber of average (KS) and poor (KG) quality
WR–A	reinforced beams made of glulam timber of average (KS) and poor quality (KG), reinforced with BFRP, reinforcement ratio: 1.18%
WR–B	reinforced beams made of glulam timber of average (KS) and poor quality (KG), reinforced with BFRP, reinforcement ratio: 1.76%

**Table 3:** Basalt fibres and epoxy glue mechanical properties (manufacturer data).

Feature	BFRP	Epoxy Glue
Materials properties	anisotropic	-
Density	1.9 ÷ 2.10 g/cm^3^	-
Tensile strength	do 1.45 GPa	-
Flexural strength	-	0.11 ÷ 0.12 GPa
Modulus of elasticity	do 78 GPa	2.7 ÷ 3.3 GPa
Linear expansion coefficient	2.2%	-

**Table 4:** Wood and FRP materials data.

Material Parameters	Young’s Modulus (GPa)			Poisson’s Ratio			Shear Modulus (GPa)		
	X Axis (the Longitudinal Direction)	Y Axis (the Radial Direction)	Z Axis (the Tangential Direction)	XY Axis (for a Radial Surface)	YZ Axis (for the Face)	XZ Axis (for the Tangent Surface)	XY Axis (for a Radial Surface)	YZ Axis (for the Face)	XZ Axis (for a Radial Surface)
Lamella KS	10	0.33	0.33	0.54	0.027	0.54	0.63	0.063	0.63
Lamella KG	7	0.23	0.23	0.54	0.027	0.54	0.44	0.044	0.44
BFRP	56.30	9.38	9.38	0.26	0.026	0.26	1.9	0.19	1.9

**Table 5:** Epoxy glue material data.

Material Parameters	Young’s Modulus (GPa)	Poisson’s Ratio
Epoxy glue	3	0.30

**Table 6:** Experimental research results, lamellas I and IV, base 7.

BEAM	F/2 [kN]	Compressive Stresses σ_c_ [MPa]	Tensile Stresses σ_t_ [MPa]	Stresses in BFRP Fibres σ_BFRP_ [MPa]	Displacements u [mm]
NWR-A1	5	−13.12	8.54	-	13.42
NWR-A2	5	−10.63	15.97	-	13.92
NWR-A3	5	−8.34	10.22	-	13.64
Mean		−10.70	11.58	-	13.66
Standard deviation		2.39	3.90	-	0.25
WR-A1	5	−12.00	13.07	58.74	12.89
WR-A2	5	−6.92	11.60	44.88	11.91
WR-A3	5	−11.90	13.38	55.69	12.09
Mean		−10.27	12.68	53.10	12.30
Standard deviation		2.90	0.95	7.28	0.52
WR-A1	10	−26.60	26.65	117.75	28.05
WR-A2	10	−17.95	22.68	97.53	27.7
WR-A3	10	−25.85	28.48	126.62	29.06
Mean		−23.47	25.94	113.97	28.27
Standard deviation		4.79	2.97	14.91	0.70

**Table 7:** Comparison of selected results obtained using the laboratory, analytical and numerical methods, lamellas I and IV, base 7.

Beam	F/2 [kN]	Experimental Results				Theoretical Results	Numerical Results			
		σ_c_ [MPa]	σ_t_ [MPa]	σ_BFRP_ [MPa]	u [mm]	σ_BFRP_ [MPa]	σ_c_ [MPa]	σ_t_ [MPa]	σ_BFRP_ [MPa]	u [mm]
NWR (Mean)	5	−10.70	11.58	-	13.66	-	−9.86	10.57	-	16.30
WR-A (Mean)	5	−10.27	12.68	53.10	12.30	82.34	−8.55	8.65	48.83	15.10
WR-A (Mean)	10	−23.47	25.94	113.97	28.27	164.68	−18.49	17.00	100.04	30.21

## Data Availability

Data sharing is not applicable to this article.
